# Country-Level Governance Indicators as Predictors of COVID-19 Morbidity, Mortality, and Vaccination Coverage: An Exploratory Global Analysis

**DOI:** 10.4269/ajtmh.22-0107

**Published:** 2022-10-31

**Authors:** Mahdi Baghbanzadeh, Madison Smith, Juergen Pilz, M. Sohel Rahman, Ajlina Karamehic-Muratovic, Ashvita Garg, Esther Annan, Uyen-Sa D T Nguyen, Nathan Schedler, Rajesh Nandy, Rafiul Islam, Ubydul Haque

**Affiliations:** ^1^ Operations, Snapp, Tehran, Iran;; ^2^ Department of Biostatistics and Epidemiology, University of North Texas Health Science Center, Fort Worth, Texas;; ^3^ Alpen-Adria University of Klagenfurt, Klagenfurt, Austria;; ^4^ Department of Computer Science and Engineering, Bangladesh University of Engineering and Technology, ECE Building, Dhaka, Bangladesh;; ^5^ Department of Sociology and Anthropology, Saint Louis University, St. Louis, Missouri;; ^6^ Doisy College of Health Sciences, Saint Louis University, St. Louis, Missouri;; ^7^ Independent Development Practitioner, Adabor, Dhaka, Bangladesh

## Abstract

As the COVID-19 pandemic continues to affect all countries across the globe, this study seeks to investigate the relationship between nations’ governance, COVID-19 national data, and nation-level COVID-19 vaccination coverage. National-level governance indicators (corruption index, voice and accountability, political stability, and absence of violence/terrorism), officially reported COVID-19 national data (cases, death, and tests per one million population), and COVID-19 vaccination coverage was considered for this study to predict COVID-19 morbidity and mortality. Results indicate a strong relationship between nations’ governance and officially reported COVID-19 data. Countries were grouped into three clusters using only the governance data: politically stable countries, average countries or “less corrupt countries,” and corrupt countries or “more corrupt countries.” The clusters were then tested for significant differences in reporting various aspects of the COVID-19 data. According to multinomial regression, countries in the cluster of politically stable nations reported significantly more deaths, tests per one million, total cases per one million, and higher vaccination coverage compared with nations both in the clusters of corrupt countries and average countries. The countries in the cluster of average nations reported more tests per one million and higher vaccination coverage than countries in the cluster of corrupt nations. Countries included in the corrupt cluster reported a lower death rate and morbidity, particularly compared with the politically stable nations cluster, a trend that can be attributed to poor governance and inaccurate COVID-19 data reporting. The epidemic evaluation indices of the COVID-19 cases demonstrate that the pandemic is still evolving on a global level.

## INTRODUCTION

The novel COVID-19 pandemic is a serious global health threat and has disrupted virtually all aspects of daily life.^1^ As of October 18, 2021, the WHO had documented 240 million total confirmed COVID-19 cases around the world, with 4.89 million confirmed deaths and a total of 6545 million vaccine doses administered.^2^^,^^3^

To combat COVID-19 at the national level, a country needs 1) a strong health infrastructure, 2) a government that can implement sound policies and regulations that promote public health, and 3) good governance that can both make informed and transparent decisions and manage risks. Political stability enables the formulation of urgent policies and guidelines needed for the control of the COVID-19 pandemic. In the absence of policy actions, it is estimated that COVID-19 infections exhibit growth rates of approximately 38% a day.^4^

COVID-19 control measures have typically included travel restrictions and quarantines. On March 17, 2020, the Schengen area closed all its external borders to control the spread of the COVID-19 pandemic.^5^ This move was intended to stop viral mobility throughout Europe, especially Central Europe, Spain, and France.^6^ In Asia, Malaysia similarly initiated a Movement Control Order on March 18, 2020, requiring the closure of all businesses except those deemed essential.^7^ This order proved to be very successful as a downward trend in COVID-19 cases became evident around mid-April. The lockdown policies initiated in Lagos, Nigeria, likewise had a strong impact in slowing down COVID-19 incidences, as the rise in the rate of new cases was reduced by a factor of 0.65.^8^ Analysis of China’s early response to the pandemic demonstrates the tension between the federal power concentration and local governance during the early phases of the epidemic, which led to a breakdown of their public health model and created a time lag between federal, regional, and local responses.^9^

Although some countries have managed to control the pandemic better than others by prioritizing certain control measures, COVID-19 is still present in almost every part of the world and continues to cripple nations such as India.^10^ The mortality rate from COVID-19 is negatively associated with governmental effectiveness.^11^ Moreover, studies have shown that the increase in COVID-19 cases is due to a country’s poor governance, increased corruption, inadequate healthcare facilities, and weak public health communication.^12^^,^^13^ The COVID-19 pandemic has tested healthcare systems and their structure for emergency responsiveness through their supply-chain response and high demand for ICU beds.^14^ Thus far, many models of governance have shown poor response, especially in rural areas where the emergency support system has suffered severely with a shortage of medical supplies and poor treatment levels.^15^

Since the onset of the pandemic, a plethora of literature has been published on COVID-19. To the best of our knowledge, however, no study has been conducted that examines the role of the regulatory quality and corruption perceptions index in the effectiveness of the control of COVID-19 in a given country. In this study, we hypothesized that countries with different levels of governance vary as to the number of COVID-19 tests, morbidity, and mortality rates reported. Thus, this study attempts to reveal the relationships among the regulatory quality, political stability, corruption perceptions index, and the effectiveness of governance to combat the COVID-19 pandemic.

## METHOD

### Governance indicator data.

Governance is defined as the foundations that an authority uses to govern a country. The foundations are multifaceted and range from the process of authority selection, surveillance, and replacement, to quality assurance in the creation and implementation of policies.^16^ The Worldwide Governance Indicators (WGI) are developed via information extracted from survey institutes, think tanks, nongovernmental organizations, international organizations, and private sectors and are used in more than 198 countries.^16^ The information from these categories is simplified into six broad sectors of governance via an unobserved components model. The WGI is meant to reflect the majority of initiatives and public responses within industrial and developing countries.^16^

The following 2019 country-level data were collected from the World Bank: 1) an estimate of control of corruption, 2) a percentile rank of control of corruption, 3) an estimate of government effectiveness, 4) an estimate of political stability and absence of violence/terrorism, 5) a percentile rank of political stability and absence of violence/terrorism, 6) an estimate of rule of law, 7) a percentile rank of the rule of law, 8) an estimate of regulatory quality, 9) a percentile rank of regulatory quality, 10) an estimate of regulatory quality, 11) a percentile rank of regulatory quality, 12) an estimate of voice and accountability, 13) a percentile rank of voice and accountability, and 14) corruption perceptions index 2019 at the country level.^17^^,^^18^ These data represent the estimates and percentile ranks of aggregate governance indicators of each country and territory. This set of variables is referred to as “set 1” throughout this paper. A summary of the types of data used in cluster analysis is presented in Table 1 in the supplemental appendix.

**Table 1:** Wilk’s lambda test for canonical dimensions

Dimension	Canonical correlation	Approximation	Df1	Df2	*P* value
1	0.8	18.43	12	365	< 0.001**
2	0.42	4.95	6	278	< 0.001**
3	0.06	0.29	2	140	0.75

**Significant at the level of 0.05.

### COVID-19 and vaccination.

The national statistics about COVID-19 tests per one million population, morbidity, and mortality until January 11, 2022, were collected from the Johns Hopkins website.^2^^,^^19^ The vaccination data until January 11, 2022, were also gathered from the *New York Times* website (https://www.nytimes.com/interactive/2021/world/covid-vaccinations-tracker.html). This set of variables is referred to as “set 2” throughout this paper.

### Data preparation.

After cross-matching the data from sets 1 and 2, correlation analyses (Pearson) were carried out on the data of 145 countries. Eight of the governance indicator variables were found to be highly correlated (Supplemental Figure 1) with the corruption perceptions index (CPI index) having correlations higher than 0.85; therefore, those governance indicator variables were removed from the analysis. Among the remaining variables, the correlations were higher than 0.9 between pairs of estimates and their percentile rank counterparts for voice and accountability, political stability, and absence of violence (Supplemental Figure S1). After dropping the highly correlated estimates in a pair, the CPI index (the score and percentile rank only differ in their range, the former is between –2.5 and 2.5 and the latter is between 0 to 100), along with percentile ranks of voice and accountability, political stability, and absence of violence/terrorism remained in set 1. To tackle the issue of skewness, we used the log transformation of the variables.

### Statistical analysis.

To determine the association between governance indicators and COVID-19 data (cases, deaths, tests, and vaccination), we used canonical correlation analysis (CCA), which was introduced by Hotelin in 1936.^20^ We also implemented the Wilks’ lambda test statistic to test the null hypothesis that there is no relationship between two sets of data Androniceanu (2020)^21^ and Cho (2019).^22^ We also used principal component analysis to visualize the dataset in two dimensions using only the first two principal components.^23^

First, to classify the selected countries based on the governance indicator variables, cluster analysis was performed using the model-based clustering algorithm.^24^ As it has been shown in Dasgupta and Raftery (1998)^25^ and Fraley and Raftery (1998)^24^ that the Bayesian information criterion is an appropriate measure for evaluating the goodness and stability of the (Gaussian) mixture type clustering, we implemented this criterion for the model selection procedure.^25^^,^^26^ The clustering procedure was done with the MCLUST package in the R programing language.^27^ The countries were classified into three groups or clusters: corrupted countries (cluster Cor-C), average countries (cluster Avg-C), and countries with a stable and effective political system (cluster Stb-C). We then used these clusters to identify significant differences in their official reported COVID-19 data. Significant differences in the reported COVID-19 data among the cluster countries were tested using nonparametric tests: Kruskal–Wallis test and Wilcoxon rank-sum test. Pairwise comparisons were conducted for these three clusters using the Wilcoxon test.

The multinomial regression model was used to assess the association between outcome and predictor/covariates. The response variable was specified as a cluster of countries (three clusters) and the predictor variables were the total number of COVID-19 cases, deaths, tests, vaccination, population density, and annual growth of (%) gross domestic product (GDP). We fitted a total of eight models (4 COVID-19 variables and two reference levels) to investigate the significance of each variable in presence of covariates (population density and GDP annual growth). Each model has only one of the set 2 variables (COVID-19 variables) and one of the clusters as the reference level. For example, one of the models has vaccination, population density, and GDP annual growth as its predictors, and a cluster of corrupted countries as the reference level. Another model has the same predictors, but its reference level is the cluster of average countries. Data analysis was performed using the “nnet” library in the statistical software R (version 4.1.2).

## RESULTS

**Figure 1.**
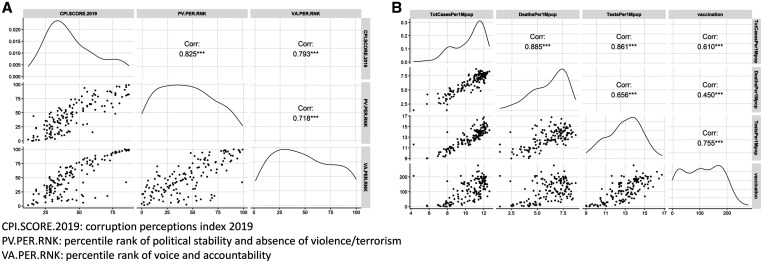
(**A**) Pair plots of the variables in set 1 (corruption characteristics variables). (**B**) Pair plots of the variables in set 2 (COVID-19 data).

**Figure 2.**
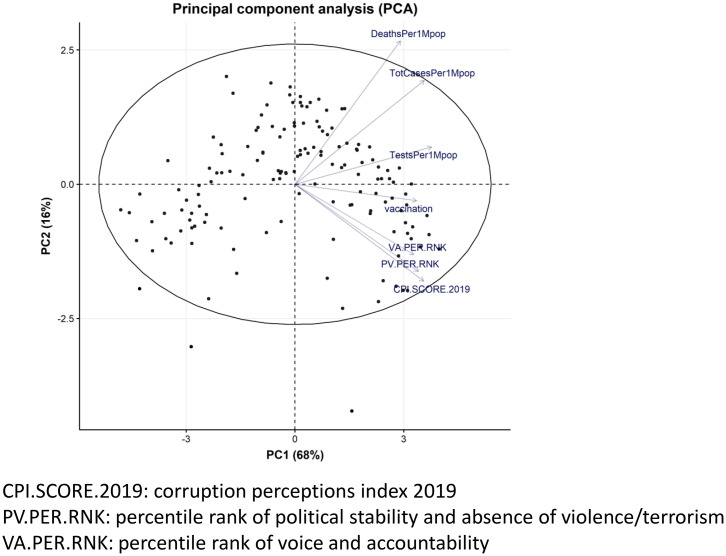
Principal components of data.

The distributions and linear relationships in both set of variables are illustrated in Figure 1, which shows that the variables were positively correlated. Among the variables in set 2, the number of tests per one million populations had significantly higher correlations with variables in set 1 (Supplemental Figure 2). The first principal component was affected positively by all the variables, and the second principal component was positively affected by all the variables in set 1 and affected negatively by all the variables in set 2 (Figure 2).

The first and the second dimensions of the canonical correlations were significant at the 0.05 level (Table 1). The first dimension of the canonical correlation between sets of variables was 0.8, and the second was 0.42. The first canonical dimension for set 1 (corruption characteristics variables) was dominated by the CPI score, and the first canonical dimension for set 2 (COVID-19 data) was mainly influenced by the number of tests per one million population (Table 2). The second dimension was dominated by the voice and accountability percentile rank, whereas it is dominated by deaths and total cases per one million population for set 2 (Table 2).

**Table 2:** Standardized canonical coefficients

		Dimensions	
		1	2
Corruption variables	CPI score*	−0.96	−0.21
	PV.PER.RNK†	−0.83	−0.38
	VA.PER.RNK‡	−0.62	−0.75
COVID-19 variables	Total cases per 1 M	−0.49	−0.32
	Deaths per 1 M	−0.24	−0.38
	Tests per 1 M	−0.72	−0.14
	Vaccination per 100 people	−0.69	−0.11

*Corruption perceptions index 2019.

† Percentile rank of political stability and absence of violence/terrorism.

‡ Percentile rank of voice and accountability.

**Figure 3.**
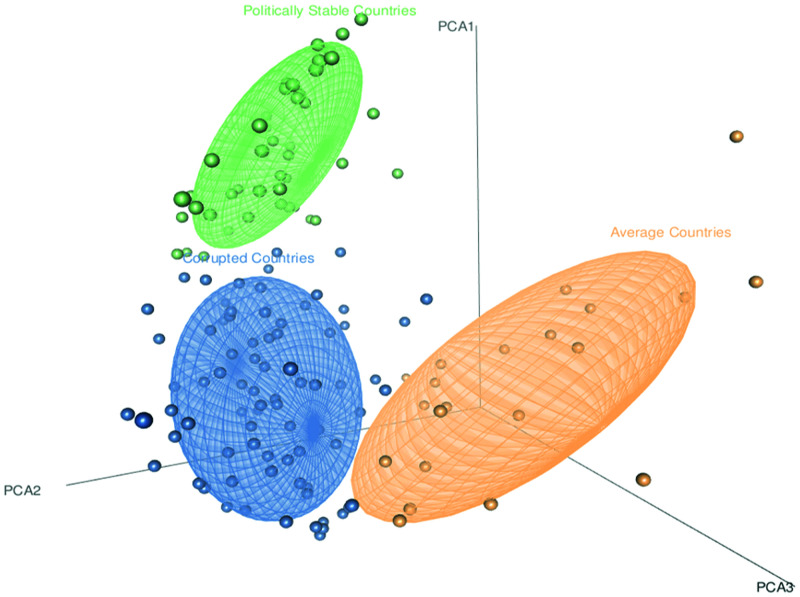
Clusters of countries based on the variables of set 1 (model-based clusters). This figure appears in color at www.ajtmh.org.

**Figure 4.**
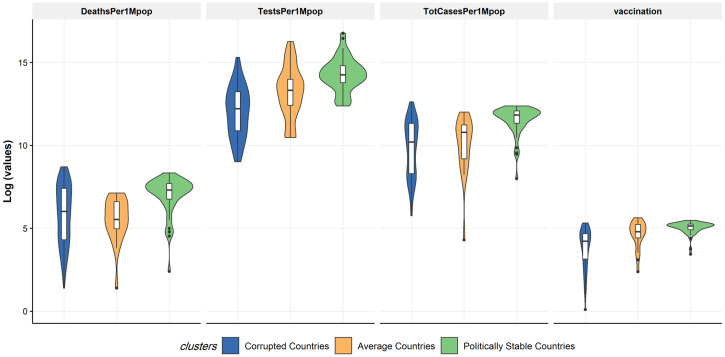
Distribution of variables in clusters (model-based clusters). This figure appears in color at www.ajtmh.org.

As indicated in Supplemental Figure 3, the cluster Cor-C (*N* = 78) has the lowest mean value in the corruption perceptions index and a percentile rank of political stability and absence of violence/terrorism, whereas the cluster of average countries (*N* = 21) had the lowest mean value in percentile rank of voice and accountability, and the cluster of politically stable countries (*N* = 46) has the highest mean value in all three variables. In Figure 3, the performance of the clustering on the first three principal components is illustrated in a 3D scatter plot, and Supplemental Figure 4 shows a world map of clusters of specific countries. Figure 4 compares the distribution of variables of set 2 (COVID-19 data) in each cluster (recall that the clusters were calculated based on the variables of set 1). Wider parts of the violins show a concentration of values for specific variables in set 2 and if values concentrate around the variable’s minimum or maximum, the violin contains a clipping in the upper or lower bounds. The cluster of politically stable countries has uniformly higher median values (midlines in box plots within violins) in all the variables in set 2. The cluster Cor-C (blue violin) has the lowest median value in all the variables except for death in one million population.

The results of the Kruskal–Wallis test showed that the number of total cases (*P* < 0.001), the number of deaths (*P* < 0.001), the number of tests (*P* < 0.001), and the rate of vaccination between clusters of countries (*P* < 0.001) were significantly different between clusters (Table 3).

**Table 3:** *P* values of Kruskal–Wallis rank-sum test

Variable	Df	Kruskal–Wallis χ^2^	*P* value
Total Cases per 1 million	2	42.9	< 0.001**
Deaths per 1 million	2	20.4	< 0.001**
Tests per 1 million	2	53.7	< 0.001**
Vaccination per 100 people	2	20.9	< 0.001**

**Significant at the level of 0.05.

The *P* values of the paired comparisons of the variables using Wilcoxon rank-sum test results are reported in Table 4. The number of tests per one million is significantly different at the level of 0.05 between all the pairs of the clusters. There is a significant difference at the level of 0.05 between clusters of corrupted and politically stable countries for all the variables in set 2. The clusters of average countries and politically stable countries also show significantly different values for total cases, deaths, and tests per one million population. The clusters of corrupted countries and average countries also have significantly different values for vaccination and tests per one million population (Table 4).

**Table 4:** *P* values of pairwise comparisons using Wilcoxon rank-sum test

		Total Cases per 1 million	Deaths per 1 million	Tests per 1 million	Vaccination per 100 people
Corrupt countries	Average countries	0.53	0.36	0.01**	0.003**
	Politically stable countries	< 0.001**	< 0.001**	< 0.001**	< 0.001**
Average countries	Politically stable countries	< 0.001**	< 0.001**	0.01**	0.05

** Significant at the level of 0.05.

The results of all the eight multinomial logistic regression models are reported in Table 4 of the supplemental appendix. According to the reference level of each model, the regression coefficients sign (positive or negative) and their *P* value (being significant or not), the following results can be inferred: There is a significant difference between countries in the cluster of politically stable countries and countries in the other two clusters in all the variables of set 2 (COVID-19 variables). Countries in the cluster of politically stable countries significantly reported more cases, deaths, tests per one million population, and a higher rate of vaccination compared with countries in the cluster of average countries and cluster of corrupted countries. Additionally, countries in the cluster of average countries significantly reported more tests in one million population and a higher rate of vaccination than those in the cluster of corrupted countries. However, there is no significant difference between the number of reported deaths between countries in the cluster of average countries and the cluster of corrupted countries.

## DISCUSSION

The COVID-19 pandemic has uncovered the likelihood of corruption through the exploitation of weak oversight and inadequate transparency of governmental COVID-19 emergency responses. With all crises, there are trade-offs between corruption prevention and enforcement mechanisms and urgent and flexible response. Natural disasters and health emergencies have historically been plagued by challenges of corruption, especially in areas of a preexisting corrupt government. This corruption derives from the loosening of procurement oversight and enforcement during the rapid mobilization of immense economic and medical resources to address public health emergencies.^28^ For example, in a study of the political influences on the response to the Ebola epidemic in Sierra Leone, it was found that a lack of a strong and resilient governmental infrastructure impeded an effective response to the public health crisis.^29^ Therefore, there is a need during the COVID-19 pandemic to understand governance indicators as predictors of the management and response to the outbreak of COVID-19 in specific countries. Moreover, understanding the role of governance indicators during COVID-19 can inform and guide sustainable recovery well past the pandemic as well as build resilience toward potential future pandemics.

Our study aimed to determine the role of the regulatory quality, political stability, corruption perceptions index, and the effectiveness of governance in mitigating and controlling the COVID-19 morbidity and mortality reported by each country throughout the pandemic. The findings of this study could inform the relationships among governmental transparency, security, and political stability in managing the COVID-19 pandemic while also providing possible guidelines to curb and mitigate future public health crises.^30^ The findings of the study can additionally help nations identify gaps that need to be filled when it comes to COVID-19 and national health security.^31^^,^^32^

One of the main study findings was that countries clustered as politically stable reported significantly higher numbers of deaths, tests, and COVID-19 cases per one million population than countries clustered as corrupt. In our study, underreporting is demonstrated by the Wilcoxon test (see Tables 3 and 4), an exact quantification of the amount of underreporting via generalized linear modeling would necessitate reliable data on fraudulent COVID-19 reporting. It may further explain, at least partially, the lower numbers of COVID-19 cases per one million in more corrupt countries compared with the countries without corruption (Figure 4, Table 4, and Table 4 in the supplemental appendix). Countries such as Mexico have reported significant delays in death reports for COVID-19.^33^^,^^34^ Low- to middle-income countries are likely to experience challenges of increased death report delays due to a lack of real-time information. Valid statistical claims would necessitate further reliable data such as numbers of people with access to testing relative to whole population numbers.

According to a study by Farzanegan et al., which analyzed the effect of corruption on COVID-19 immunization, corruption disrupts the enforcement of vaccination by increasing public distrust of government.^35^ The high-income inequality found in corrupt countries can also increase the population of individuals paying for disease prevention and treatment without insurance, which creates a financial burden and barrier to combating the disease.^35^ Thus, the measures and means developed to combat the disease would not have the same magnitude of success in corrupted countries. In particular, corruption widens health inequalities and exacerbates health disparities, meaning those most vulnerable are usually the ones to suffer.

Likewise, our study showed that countries in the cluster of corrupt countries reported a significantly smaller number of tests of COVID-19 per one million than countries with a stable and effective political system (Figure 4, Table 4, and Table 4 of the supplemental appendix). If individuals do not have the means for and access to COVID-19 testing, this may significantly decrease the COVID-19 case count within the country.

Another possible reason for the significant relationship between corruption and testing rates could be corruption leading to a lack of supplies for testing. An example of this relationship is observed in the case of Indonesia.^36^ The worsening political corruption in Indonesia is one of the factors that, compared with most other states in Southeast Asia, has significantly decreased its ability to respond to the COVID-19 outbreak. After taking power, current President Joko Widodo (Jokowi) lessened the budget for the public health sector, resulting in low-quality equipment and services. Because of the lack of public health support and political corruption that siphoned supplies from the healthcare system, Indonesia had the lowest level of per capita COVID-19 testing in Southeast Asia.^36^

The results of this study ultimately align with prior research. An empirical analysis by Vadlamannati Cooray and De Soysa of health-system equity, egalitarian democracy, and COVID-19, concluded that broad egalitarian governance shows low testing rates and higher death rates despite equitable access to healthcare producing opposite results.^37^ Ultimately, they presumed that the capacity of health systems may have had a greater influence on success in combating the COVID-19 pandemic than respective certitude in government. The results of this study, however, look at specific government structures as an influence in COVID-19 reporting rather than the corruption within reporting.

There was widespread corruption in many countries. For example, according to the 2019s CPI, countries such as China, India, Gabon, Pakistan, Nigeria/Bangladesh, Central African Republic, Chad, Republic of Congo, Equatorial Guinea, Venezuela, and Somalia/South Sudan occupied the 78th, 86th, 123rd, 124th, 146th, 153rd, 162nd, 165th, 173rd, 176th, and 179th positions out of 198 countries, respectively.^18^ It was also reported that not only has investment in the public health system been limited in most of these countries clustered as corrupt, but there has also been a decrease in health expenditures as a percentage of government spending.^38^ Another explanation that can partially contribute to explaining why countries clustered as politically stable reported significantly higher numbers of deaths, tests, and COVID-19 cases per one million population than countries clustered as corrupt is that many democracies have responded differently to COVID-19 despite being politically stable. The pandemic has been noted to have exposed the weaknesses of many nations whereby some democratic nations have had a challenging time implementing COVID-19–related restrictions. The United States serves as an example of this where the pandemic has been heavily politicized, with the seriousness of the pandemic initially downplayed, and misinformation spread fast and wide.^39^ In countries like the United States, the pandemic challenged individuality, freedom, and rights, thereby arguably increasing the number of COVID-19-deaths, tests, and cases.

The topic of the ability of autocracies or democracies to better combat pandemics is controversial. The Chinese government successfully decreased the spread of COVID-19 but failed to prevent the virus from becoming a pandemic.^40^ The lack of pandemic preparedness in the United States was in opposition to the attitude of governments in Taiwan and South Korea, which conducted extensive testing and practiced self-isolation while also keeping their economies afloat.^41^ The U.S. Trump administration chastised China for its inability to contain the virus, yet its own government had similar shortcomings. For example, many people in U.S. leadership positions and certain segments of the U.S. population think that the COVID-19 pandemic is a hoax and did not take the risk of COVID-19 infection and spread in the United States seriously. In addition, they did not believe in masking or social distancing as good preventive measures and believed that individual rights should supersede that of public health concerns. Thus, the United States did not adequately prepare for or respond to the pandemic.^40^ Taiwan and South Korea demonstrated the ability of democracies to respond effectively to epidemics with proper leadership and the capacity to implement solutions.^40^

The COVID-19 pandemic caused some countries to exhibit authoritarian acts to respond to the infection.^42^ With the core of democracy being a lack of state intervention in citizens’ lives, democracy has been challenged in the times of this pandemic as opposed to its authoritarian counterparts. Authoritarian countries have had lower COVID-19 death counts compared with democratic countries. This does not necessarily infer that government intervention is vital to combat the pandemic, but rather that higher COVID-19 test rates; the ability to meet demands for increased morbidity from COVID-19; as well as cooperation in social distancing, masking, or quarantines as necessary are more appropriate indicators of success in the pandemic.^43^ Therefore, it is a delicate balance between individual liberties and governmental mandates to keep citizens safe while controlling the spread of COVID-19 infections and deaths.

Nations around the world are continuing to struggle to respond to the pandemic efficiently and rapidly. In addition to issues directly related to healthcare systems and public service delivery, there are issues related to governance and corruption as well. Curbing government corruption is beyond the scope of this study, it does shed light on the fact that most governments’ plans for responding to public health crises do not focus enough on preventing corruption and governance. This study illustrates that weak governance and increased corruption are associated with decreased reporting of cases, deaths, and testing, which may result in an inadequate or a slower and less efficient pandemic response. USAID offers several approaches to address corruption in this context and focuses on “systemic and holistic interventions that bolster the ability of countries to detect, prevent, investigate and sanction corruption across the COVID-19 response.”^44^ Government- and country-level efforts and reforms to curb corruption instituted at both local and national levels are much needed. Likewise, countries need to consider governance and prevention of corruption as a part of their response plan for future public health crises. COVID-19 pandemic will shed renewed light on governance as the pandemic continues to cost lives and weaken economies across the world.

Limitations of this study include sample size and data collection. The proportion of each cluster of politically stable, corrupt, and average countries was unevenly distributed. Second, these data were provided by individual countries and collated by the World Bank; thus, data could be intentionally inaccurate, particularly if from corrupt governments. Corruption is highly relevant in data compilation and reporting, but it is only one of the various relevant factors that can affect data quality. Corruption may manifest in various ways, each having different effects on how the data are skewed. Also, there may be alternative explanations for lower rates of deaths, cases, and testing per one million. Given that poverty and lack of infrastructure are correlated with governmental corruption, they may also explain lower testing and accurate counting.

Although governance is an important factor in influencing a country’s success in combating the pandemic, socioeconomic status is also insightful as it helps policymakers to develop strategies and measures to protect their country currently and in the future.^45^ When a country healthcare system is at maximum capacity (e.g., Bosnia, Pakistan, India), the legitimacy of the government is challenged (e.g., Egypt), and with trust issues between the government and other actors,^46^ finding the true values of COVID-19 statistics will be challenging.

COVID-19 data in some countries around the world have been misrepresented and underreported. This is particularly true in corrupt countries where governments have not always acted transparently and have tried to use the pandemic as an opportunity to fuel political agendas. Practices of corruption related to the pandemic include covering up the reality of the pandemic, especially mortality rates, to serve political agendas; malpractice when it comes to vaccines; and, quite simply, false data, among other factors. One such example is Zambia.^47^ However, because WHO excess data is used, the results of our study account for this shortcoming.

## CONCLUSION

A country’s success in halting the progression of a pandemic depends on factors such as its system of government, corruption, and transparency. A perceived low death rate in defined clustering of corrupt countries, relative to politically stable countries, may indicate a lack of resources, delays in reporting, or deliberate attempts to conceal actual death reports. The epidemic evaluation indices of COVID-19 currently show that the pandemic is still evolving and that the peak in the pandemic has not been reached. The pandemic and the response to it are already putting pressure on governments, economies, and households. In addition to providing testing and medical treatment, countries need to reduce corrupt practices and strengthen their public health infrastructure to successfully end the COVID-19 pandemic.

## Supplemental files


Supplemental materials
