# Supplementary material for: Country-Level Governance Indicators as Predictors of COVID-19 Morbidity, Mortality, and Vaccination Coverage: An Exploratory Global Analysis
Source: Am J Trop Med Hyg. 2022 Oct 31;107(5):1066–73. doi: 10.4269/ajtmh.22-0107 (PMC9709024; doi:10.4269/ajtmh.22-0107)
Supplement: Supplementary file 1 [file tpmd220107.SD1.pdf]

Table 1 (appendix): List of variables

Variable code	Variable Name	Long Definition
CC.EST	Control of Corruption: Estimate	Control of Corruption captures perceptions of the extent to which public power is exercised for private gain, including both petty and grand forms of corruption, as well as "capture" of the state by elites and private interests. Estimate gives the country's score on the aggregate indicator, in units of a standard normal distribution, i.e. ranging from approximately -2.5 to 2.5.
CC.PER.RNK	Control of Corruption: Percentile Rank	Control of Corruption captures perceptions of the extent to which public power is exercised for private gain, including both petty and grand forms of corruption, as well as "capture" of the state by elites and private interests. Percentile rank indicates the country's rank among all countries covered by the aggregate indicator, with 0 corresponding to lowest rank, and 100 to highest rank. Percentile ranks have been adjusted to correct for changes over time in the composition of the countries covered by the WGI.
GE.EST	Government Effectiveness: Estimate	Government Effectiveness captures perceptions of the quality of public services, the quality of the civil service and the degree of its independence from political pressures, the quality of policy formulation and implementation, and the credibility of the government's commitment to such policies. Estimate gives the country's score on the aggregate indicator, in units of a standard normal distribution, i.e. ranging from approximately -2.5 to 2.5.
GE.PER.RNK	Government Effectiveness: Percentile Rank	Government Effectiveness captures perceptions of the quality of public services, the quality of the civil service and the degree of its independence from political pressures, the quality of policy formulation and implementation, and the credibility of the government's commitment to such policies. Percentile rank indicates the country's rank among all countries covered by the aggregate indicator, with 0 corresponding to lowest rank, and 100 to highest rank. Percentile ranks have been adjusted to correct for changes over time in the composition of the countries covered by the WGI.
PV.EST	Political Stability and Absence of Violence/Terrorism: Estimate	Political Stability and Absence of Violence/Terrorism measures perceptions of the likelihood of political instability and/or politically-motivated violence, including terrorism. Estimate gives the country's score on the aggregate indicator, in units of a standard normal distribution, i.e. ranging from approximately -2.5 to 2.5.
PV.PER.RNK	Political Stability and Absence of Violence/Terrorism: Percentile Rank	Political Stability and Absence of Violence/Terrorism measures perceptions of the likelihood of political instability and/or politically-motivated violence, including terrorism. Percentile rank indicates the country's rank among all countries covered by the aggregate indicator, with 0 corresponding to lowest rank, and 100 to highest rank. Percentile ranks have been adjusted to correct for changes over time in the composition of the countries covered by the WGI.
RL.EST	Rule of Law: Estimate	Rule of Law captures perceptions of the extent to which agents have confidence in and abide by the rules of society, and in particular the quality of contract enforcement, property rights, the police, and the courts, as well as the likelihood of crime and violence. Estimate gives the country's score on the aggregate

		indicator, in units of a standard normal distribution, i.e. ranging from approximately -2.5 to 2.5.
RL.PER.RNK	Rule of Law: Percentile Rank	Rule of Law captures perceptions of the extent to which agents have confidence in and abide by the rules of society, and in particular the quality of contract enforcement, property rights, the police, and the courts, as well as the likelihood of crime and violence. Percentile rank indicates the country's rank among all countries covered by the aggregate indicator, with 0 corresponding to lowest rank, and 100 to highest rank. Percentile ranks have been adjusted to correct for changes over time in the composition of the countries covered by the WGI.
RQ.EST	Regulatory Quality: Estimate	Regulatory Quality captures perceptions of the ability of the government to formulate and implement sound policies and regulations that permit and promote private sector development. Estimate gives the country's score on the aggregate indicator, in units of a standard normal distribution, i.e. ranging from approximately -2.5 to 2.5.
RQ.PER.RNK	Regulatory Quality: Percentile Rank	Regulatory Quality captures perceptions of the ability of the government to formulate and implement sound policies and regulations that permit and promote private sector development. Percentile rank indicates the country's rank among all countries covered by the aggregate indicator, with 0 corresponding to lowest rank, and 100 to highest rank. Percentile ranks have been adjusted to correct for changes over time in the composition of the countries covered by the WGI.
VA.EST	Voice and Accountability: Estimate	Voice and Accountability captures perceptions of the extent to which a country's citizens are able to participate in selecting their government, as well as freedom of expression, freedom of association, and a free media. Estimate gives the country's score on the aggregate indicator, in units of a standard normal distribution, i.e. ranging from approximately -2.5 to 2.5.
VA.PER.RNK	Voice and Accountability: Percentile Rank	Voice and Accountability captures perceptions of the extent to which a country's citizens are able to participate in selecting their government, as well as freedom of expression, freedom of association, and a free media. Percentile rank indicates the country's rank among all countries covered by the aggregate indicator, with 0 corresponding to lowest rank, and 100 to highest rank. Percentile ranks have been adjusted to correct for changes over time in the composition of the countries covered by the WGI.

Table 2 (appendix): Clusters' Summary (Mean Values and Number of Countries in each Clusters)

Cluster	CPI.SCORE.2019$^{\alpha}$	PV.PER.RNK$^{\beta}$	VA.PER.RNK $^{\gamma}$	Size
Corrupted Countries	32.6	27.2	38.1	78
Average Countries	43.5	43.7	14.4	21
Politically Stable Countries	65.4	74.3	83.6	46

$^{\alpha}$: corruption perceptions index 2019

$^{\beta}$: percentile rank of political stability and absence of violence/terrorism

$^{\gamma}$: percentile rank of voice and accountability

Table 3 (appendix): List of countries

Country Name	clusters	Tot Cases Per1M pop	DeathsPer1 Mpop	TestsPer1 Mpop	CPI.SCORE .2019	PV.PER. RNK	VA.PER. RNK
Afghanistan	Corrupted Countries	7.3	4.2	9.2	16	0.5	20.7
Albania	Corrupted Countries	10.7	6.7	12.2	35	58.6	53.2
Angola	Corrupted Countries	6.6	2.9	9.5	26	34.8	22.7
Argentina	Corrupted Countries	11.0	7.2	12.3	45	46.7	67.0
Armenia	Corrupted Countries	11.1	7.2	12.6	42	30.5	40.4
Australia	Politically Stable Countries	7.0	3.6	13.4	77	82.9	95.6
Austria	Politically Stable Countries	11.1	7.0	14.9	77	81.0	93.1
Azerbaijan	Average Countries	10.3	6.0	12.6	30	21.0	7.9
Bahamas	Politically Stable Countries	10.1	6.2	12.2	64	78.6	73.4
Bahrain	Average Countries	11.4	5.8	14.6	42	17.1	10.8
Bangladesh	Corrupted Countries	8.4	4.1	10.3	26	13.8	27.6
Barbados	Politically Stable Countries	9.5	5.0	13.1	62	81.4	84.2
Belarus	Average Countries	10.5	5.5	13.3	45	58.1	11.8
Belgium	Politically Stable Countries	11.3	7.6	13.8	75	59.5	94.1
Benin	Corrupted Countries	6.4	2.2	10.7	41	41.9	57.6
Bolivia	Corrupted Countries	10.1	7.0	11.4	31	38.1	44.3
Bosnia and Herzegovina	Corrupted Countries	11.0	7.8	12.5	36	31.0	37.4
Botswana	Politically Stable Countries	9.8	5.6	12.9	61	83.3	62.6

Brazil	Corrupted Countries	11.1	7.4	11.8	35	31.9	60.6
Bulgaria	Corrupted Countries	10.9	7.7	12.7	43	60.5	59.1
Burundi	Corrupted Countries	5.6	0.4	8.9	19	7.6	5.4
Cameroon	Corrupted Countries	7.7	3.6	10.7	25	8.6	18.2
Canada	Politically Stable Countries	10.3	6.4	13.6	77	84.8	96.1
Central African Republic	Corrupted Countries	7.1	2.8	9.0	25	2.9	14.8
Chile	Politically Stable Countries	10.9	7.2	13.3	67	61.4	82.3
China	Average Countries	4.2	1.4	11.6	41	36.7	8.9
Colombia	Corrupted Countries	10.8	7.2	12.5	37	17.6	52.7
Costa Rica	Politically Stable Countries	10.7	6.4	12.1	56	62.4	84.7
Croatia	Politically Stable Countries	11.2	7.4	12.9	47	73.8	64.5
Cuba	Average Countries	9.0	3.8	12.6	48	67.6	9.9
Cyprus	Politically Stable Countries	10.7	5.4	15.0	58	64.8	80.8
Czech Republic	Politically Stable Countries	11.9	7.9	14.2	56	87.1	78.3
Denmark	Politically Stable Countries	10.6	6.0	15.5	87	82.4	98.0
Djibouti	Average Countries	9.2	4.7	11.9	30	41.4	11.3
Dominican Republic	Corrupted Countries	10.1	5.7	11.7	28	47.6	51.2
Ecuador	Corrupted Countries	9.9	6.9	11.1	38	43.3	47.3
Egypt	Average Countries	7.6	4.8	10.2	35	11.9	13.3
El Salvador	Corrupted Countries	9.2	5.8	11.8	34	33.3	48.3

Equatorial Guinea	Average Countries	8.5	4.3	11.3	16	43.8	2.5
Eshwatini	Average Countries	9.7	6.4	11.9	34	35.2	12.3
Estonia	Politically Stable Countries	11.4	6.7	13.7	74	66.2	89.7
Ethiopia	Average Countries	7.6	3.4	10.0	37	9.5	16.3
Finland	Politically Stable Countries	9.6	5.1	13.5	86	81.9	98.5
France	Politically Stable Countries	11.3	7.3	13.9	69	51.9	88.2
Gabon	Corrupted Countries	9.2	4.1	12.6	31	37.6	22.2
Gambia	Corrupted Countries	7.7	4.2	10.1	37	44.8	38.4
Georgia	Corrupted Countries	11.2	6.9	13.8	56	30.0	56.2
Germany	Politically Stable Countries	10.5	6.9	13.3	80	66.7	95.1
Ghana	Corrupted Countries	8.0	3.2	10.4	41	47.1	68.0
Greece	Politically Stable Countries	10.3	6.8	13.5	48	50.0	75.4
Guatemala	Corrupted Countries	9.3	6.0	11.0	26	27.1	35.5
Guinea	Corrupted Countries	7.4	2.4	10.3	29	16.7	26.1
Guinea-Bissau	Corrupted Countries	7.5	3.5	10.3	18	21.9	29.1
Guyana	Corrupted Countries	9.6	5.8	11.8	40	41.0	56.7
Haiti	Corrupted Countries	7.0	3.1	8.6	18	23.3	26.6
Honduras	Corrupted Countries	9.9	6.2	10.9	26	26.7	31.5
Hungary	Politically Stable Countries	11.2	7.8	13.2	44	73.3	58.6
Iceland	Politically Stable Countries	9.8	4.5	14.3	78	96.7	94.6
India	Corrupted Countries	9.2	4.8	12.1	41	14.8	60.1

Indonesia	Corrupted Countries	8.7	5.1	10.8	40	27.6	52.2
Iraq	Corrupted Countries	10.1	5.9	12.3	20	1.4	21.2
Ireland	Politically Stable Countries	10.8	6.9	13.7	74	86.2	92.1
Israel	Corrupted Countries	11.4	6.5	14.2	60	15.2	70.0
Italy	Politically Stable Countries	11.1	7.6	13.7	53	57.6	81.8
Ivory Coast	Corrupted Countries	7.4	2.4	9.9	35	15.7	37.9
Jamaica	Politically Stable Countries	9.6	5.5	11.6	43	62.9	69.0
Japan	Politically Stable Countries	8.3	4.3	11.3	73	88.1	80.3
Jordan	Average Countries	11.1	6.7	13.3	48	31.4	28.1
Kazakhstan	Average Countries	9.6	5.2	13.1	34	45.7	15.8
Kenya	Corrupted Countries	7.9	3.8	10.3	28	12.4	35.0
Kuwait	Corrupted Countries	11.0	5.8	13.1	40	52.4	30.5
Kyrgyzstan	Corrupted Countries	9.5	5.5	11.8	30	25.2	34.0
Latvia	Politically Stable Countries	11.0	7.0	13.9	56	61.0	74.9
Lebanon	Corrupted Countries	11.2	6.9	13.2	28	7.1	32.0
Libya	Corrupted Countries	10.1	6.0	11.8	18	1.9	6.9
Lithuania	Politically Stable Countries	11.4	7.2	13.8	60	72.9	77.8
Luxembourg	Politically Stable Countries	11.5	7.1	15.2	80	96.2	96.6
Madagascar	Corrupted Countries	6.9	2.9	8.6	24	28.1	36.5
Malawi	Corrupted Countries	7.5	4.1	9.4	31	34.3	42.4
Malaysia	Corrupted Countries	9.3	3.7	12.5	53	54.3	41.4

Maldives	Corrupted Countries	10.8	4.8	14.0	29	50.5	30.0
Mali	Corrupted Countries	6.4	3.0	9.4	29	5.2	36.9
Malta	Politically Stable Countries	11.1	6.8	14.5	54	93.8	83.7
Mauritania	Corrupted Countries	8.2	4.6	10.7	28	22.4	24.1
Mauritius	Politically Stable Countries	6.9	2.6	12.5	52	79.0	72.4
Mexico	Corrupted Countries	9.8	7.4	10.8	29	25.7	45.8
Moldova	Corrupted Countries	11.0	7.2	12.3	32	32.9	40.9
Montenegro	Corrupted Countries	11.9	7.7	13.2	45	51.0	50.2
Morocco	Corrupted Countries	9.5	5.5	12.0	41	33.8	29.6
Mozambique	Corrupted Countries	7.7	3.3	9.7	26	19.0	32.5
Myanmar	Corrupted Countries	7.9	4.1	10.8	29	10.5	23.6
Namibia	Politically Stable Countries	9.8	5.4	11.9	52	68.1	63.1
Nepal	Corrupted Countries	9.2	4.7	11.3	34	23.8	39.4
Netherlands	Politically Stable Countries	11.3	6.9	13.4	82	78.1	97.0
New Zealand	Politically Stable Countries	6.3	1.8	12.9	87	99.0	99.5
Niger	Corrupted Countries	5.3	2.2	8.2	32	11.4	31.0
Nigeria	Corrupted Countries	6.7	2.4	9.1	26	4.3	33.5
North Macedonia	Corrupted Countries	11.1	7.6	12.7	35	39.5	45.3
Norway	Politically Stable Countries	9.9	4.9	13.7	84	90.5	100.0
Oman	Average Countries	10.4	5.9	12.6	52	68.6	19.7
Pakistan	Corrupted Countries	8.1	4.3	10.8	32	3.3	25.6

Panama	Politically Stable Countries	11.3	7.3	13.2	36	56.2	68.5
Papua New Guinea	Corrupted Countries	6.9	2.2	9.0	28	22.9	48.8
Paraguay	Corrupted Countries	10.4	6.6	11.8	28	42.4	49.3
Peru	Corrupted Countries	10.8	7.4	12.6	36	37.1	55.2
Philippines	Corrupted Countries	9.0	5.0	11.5	34	12.9	47.8
Poland	Politically Stable Countries	11.1	7.4	12.8	58	65.7	71.9
Portugal	Politically Stable Countries	11.3	7.4	13.8	62	89.5	88.7
Qatar	Average Countries	11.1	4.8	13.4	62	69.0	14.3
Romania	Corrupted Countries	10.9	7.2	12.9	44	48.6	61.6
Russia	Corrupted Countries	10.4	6.6	13.7	28	29.0	19.2
Rwanda	Average Countries	7.5	3.2	11.4	53	52.9	17.2
Sao Tome and Principe	Corrupted Countries	9.2	5.1	10.9	46	64.3	51.7
Saudi Arabia	Average Countries	9.3	5.3	13.0	53	28.6	5.9
Senegal	Corrupted Countries	7.7	4.2	10.2	45	42.9	55.7
Serbia	Corrupted Countries	11.2	6.5	12.9	39	49.5	46.3
Singapore	Average Countries	9.2	1.8	14.2	85	98.6	41.9
Slovakia	Politically Stable Countries	11.1	7.6	13.0	50	72.4	76.8
Slovenia	Politically Stable Countries	11.6	7.6	13.2	60	80.0	79.3
South Africa	Corrupted Countries	10.2	6.8	12.0	44	36.2	70.4
South Korea	Politically Stable Countries	7.7	3.6	12.0	59	65.2	73.9
South Sudan	Corrupted Countries	6.8	2.4	9.4	12	2.4	1.5

Spain	Politically Stable Countries	11.2	7.4	13.8	62	55.2	82.8
Sri Lanka	Corrupted Countries	8.4	3.4	11.6	38	40.5	46.8
Sudan	Corrupted Countries	6.6	3.9	8.6	16	5.7	3.0
Suriname	Corrupted Countries	9.7	5.7	11.3	44	49.0	58.1
Sweden	Politically Stable Countries	11.4	7.2	13.6	85	80.5	97.5
Switzerland	Politically Stable Countries	11.2	7.1	13.5	85	95.2	99.0
Thailand	Average Countries	6.3	0.7	11.7	36	19.5	20.2
Togo	Corrupted Countries	7.3	2.7	10.5	29	14.3	27.1
Trinidad and Tobago	Politically Stable Countries	8.7	4.7	11.3	40	54.8	67.5
Tunisia	Corrupted Countries	10.1	6.7	11.5	43	16.2	53.7
Turkey	Corrupted Countries	10.8	6.0	13.1	39	10.0	25.1
Uganda	Corrupted Countries	6.8	2.1	9.9	28	21.4	28.6
Ukraine	Corrupted Countries	10.7	6.8	12.2	30	6.2	44.8
United Arab Emirates	Average Countries	10.8	5.0	15.2	71	71.4	17.7
United Kingdom	Politically Stable Countries	11.1	7.5	14.5	77	48.1	93.6
United States	Politically Stable Countries	11.5	7.5	14.1	69	61.9	81.3
Uruguay	Politically Stable Countries	10.7	6.2	13.0	71	87.6	89.2
Uzbekistan	Average Countries	7.8	3.0	10.6	25	35.7	6.4
Venezuela	Corrupted Countries	8.7	4.2	11.7	16	9.0	10.3
Vietnam	Average Countries	3.4	0.3	10.2	37	53.8	9.4
Yemen	Corrupted Countries	5.2	3.6	6.6	15	0.0	3.9

Zambia	Corrupted Countries	8.5	4.2	11.2	34	53.3	36.0
Zimbabwe	Corrupted Countries	7.8	4.6	10.2	24	20.5	16.7

Table 4 (appendix): Multinomial Logistic Regression

avgC	**Cluster**	**Intercept**	**Death per 1 M**	**Population Density**	**GDP**
Coefficients	Corrupted Countries	2.45	0.05	-0.31	-0.32
	Politically Stable Countries	-1.99	0.62	-0.25	-0.35
Std. Errors	Corrupted Countries	1.32	0.15	0.19	0.27
	Politically Stable Countries	1.70	0.20	0.21	0.29
Z	Corrupted Countries	1.86	0.36	-1.65	-1.19
	Politically Stable Countries	-1.17	3.09	-1.21	-1.20
P-Value	Corrupted Countries	0.064	0.721	0.099	0.232
	Politically Stable Countries	0.242	0.002**	0.227	0.228

corC	**Cluster**	**Intercept**	**Death per 1 M**	**Population Density**	**GDP**
Coefficients	Average Countries	-2.45	-0.05	0.31	0.32
	Politically Stable Countries	-4.44	0.57	0.06	-0.03
Std. Errors	Average Countries	1.32	0.15	0.19	0.27
	Politically Stable Countries	1.31	0.16	0.14	0.21
Z	Average Countries	-1.86	-0.36	1.65	1.19
	Politically Stable Countries	-3.40	3.51	0.38	-0.13
P-Value	Average Countries	0.064	0.721	0.099	0.232
	Politically Stable Countries	0.001**	0.000**	0.702	0.897

avgC	**Cluster**	**Intercept**	**Tests per 1 M**	**Population Density**	**GDP**
Coefficients	Corrupted Countries	11.03	-0.64	-0.36	-0.57
	Politically Stable Countries	-6.83	0.68	-0.39	-0.51
Std. Errors	Corrupted Countries	2.79	0.20	0.21	0.29
	Politically Stable Countries	3.34	0.24	0.21	0.30
Z	Corrupted Countries	3.96	-3.24	-1.72	-1.98
	Politically Stable Countries	-2.04	2.83	-1.89	-1.72
P-Value	Corrupted Countries	0.000**	0.001**	0.085	0.048
	Politically Stable Countries	0.041**	0.005**	0.059	0.086

CorC	**Cluster**	**Intercept**	**Tests per 1 M**	**Population Density**	**GDP**
Coefficients	Average Countries	-11.03	0.64	0.36	0.57
	Politically Stable Countries	-17.87	1.32	-0.03	0.06
Std. Errors	Average Countries	2.79	0.20	0.21	0.29
	Politically Stable Countries	3.14	0.23	0.17	0.26
Z	Average Countries	-3.96	3.24	1.72	1.98
	Politically Stable Countries	-5.68	5.75	-0.19	0.22
P-Value	Average Countries	0.000**	0.001**	0.085	0.048
	Politically Stable Countries	0.000**	0.000**	0.849	0.828

avgC	Cluster	Intercept	Total Cases per 1 M	Population Density	GDP
Coefficients	Corrupted Countries	4.48	-0.16	-0.32	-0.45
	Politically Stable Countries	-9.31	1.05	-0.32	-0.30
Std. Errors	Corrupted Countries	1.92	0.16	0.19	0.28
	Politically Stable Countries	3.23	0.28	0.21	0.27
Z	Corrupted Countries	2.33	-1.01	-1.70	-1.64
	Politically Stable Countries	-2.88	3.77	-1.53	-1.12
P-Value	Corrupted Countries	0.020**	0.313	0.088	0.101
	Politically Stable Countries	0.004**	0.000**	0.126	0.264

CorC	Cluster	Intercept	Total Cases per 1 M	Population Density	GDP
Coefficients	Average Countries	-4.48	0.16	0.32	0.45
	Politically Stable Countries	-13.79	1.22	0.00	0.15
Std. Errors	Average Countries	1.92	0.16	0.19	0.28
	Politically Stable Countries	2.91	0.26	0.15	0.22
Z	Average Countries	-2.33	1.01	1.70	1.64
	Politically Stable Countries	-4.73	4.75	0.00	0.66
P-Value	Average Countries	0.020**	0.313	0.088	0.101
	Politically Stable Countries	0.000**	0.000**	0.998	0.509

avgC	Cluster	Intercept	Vaccination	Population Density	GDP
Coefficients	Corrupted Countries	6.20	-0.86	-0.25	-0.43
	Politically Stable Countries	-6.23	1.86	-0.44	-0.61
Std. Errors	Corrupted Countries	1.69	0.34	0.20	0.26
	Politically Stable Countries	2.98	0.62	0.21	0.31
Z	Corrupted Countries	3.66	-2.54	-1.24	-1.68
	Politically Stable Countries	-2.09	3.01	-2.09	-1.95
P-Value	Corrupted Countries	0.000**	0.011**	0.215	0.094
	Politically Stable Countries	0.036**	0.003**	0.036	0.051

CorC	Cluster	Intercept	Vaccination	Population Density	GDP
Coefficients	Average Countries	-6.20	0.86	0.25	0.43
	Politically Stable Countries	-12.42	2.72	-0.19	-0.18
Std. Errors	Average Countries	1.69	0.34	0.20	0.26
	Politically Stable Countries	2.72	0.57	0.16	0.26
Z	Average Countries	-3.66	2.54	1.24	1.68
	Politically Stable Countries	-4.57	4.73	-1.16	-0.69
P-Value	Average Countries	0.000**	0.011**	0.215	0.094
	Politically Stable Countries	0.000**	0.000**	0.247	0.493

**: significant at level 0.05

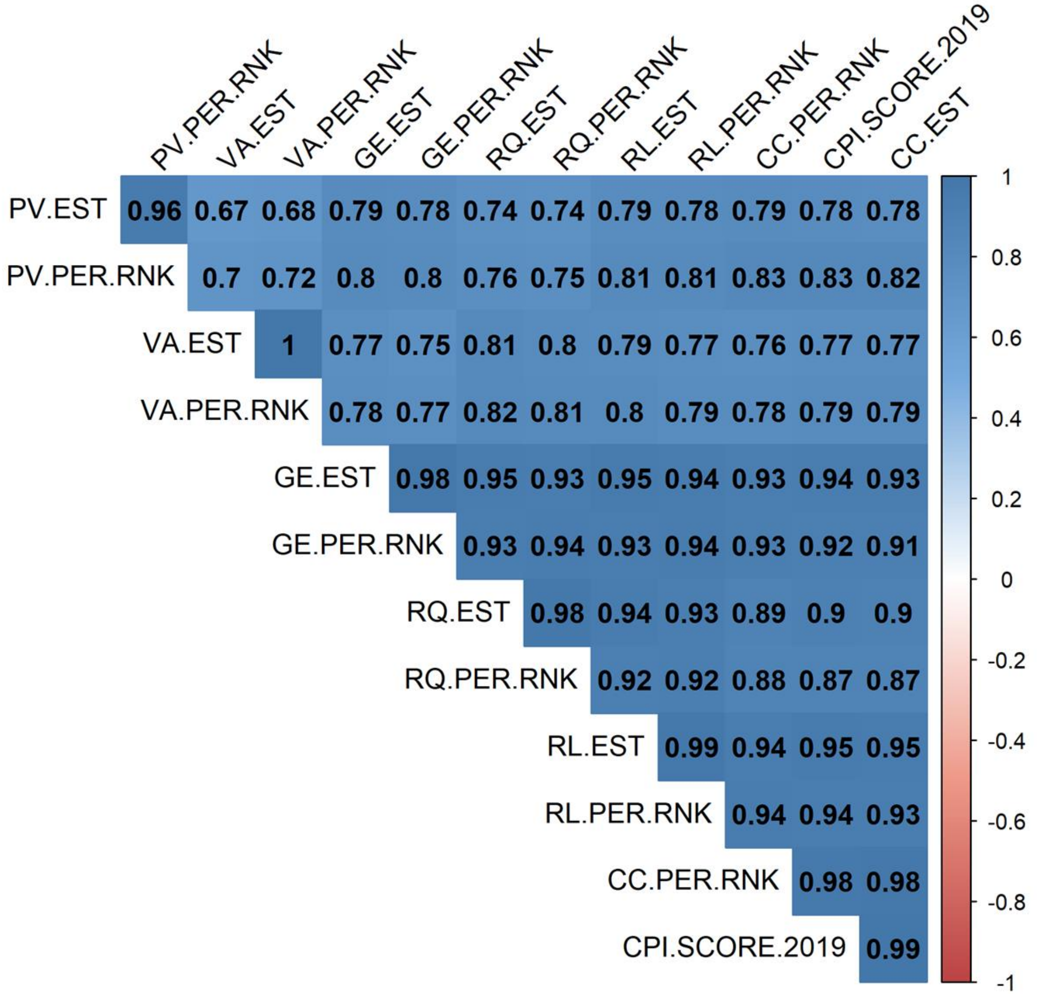

Figure S1: Correlations Between Variables of Set 1 (Corruption Characteristics Variables)

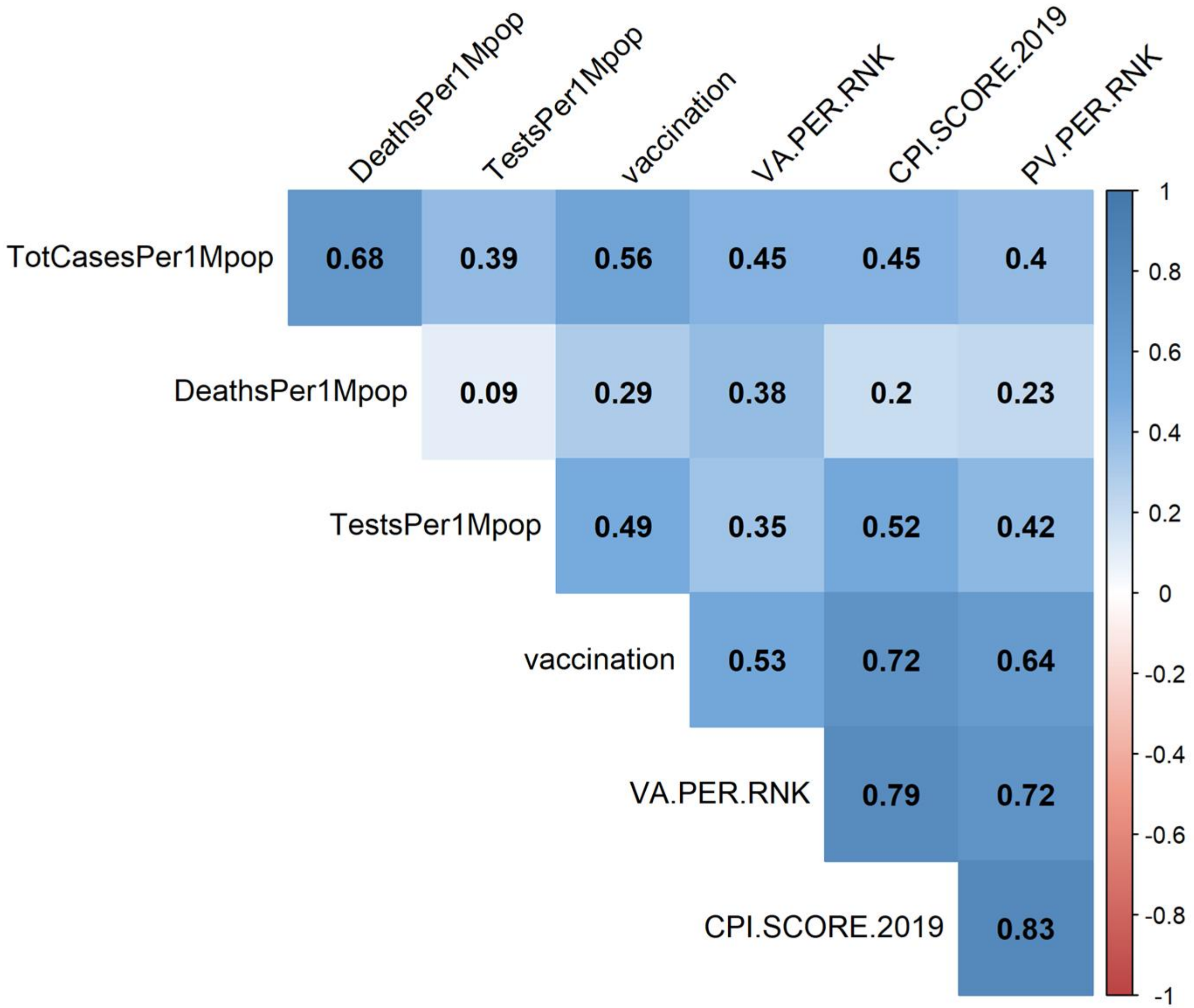

CPI.SCORE.2019: corruption perceptions index 2019
PV.PER.RNK: percentile rank of political stability and absence of violence/terrorism
VA.PER.RNK: percentile rank of voice and accountability

Figure S2: Correlation plots of the Variables

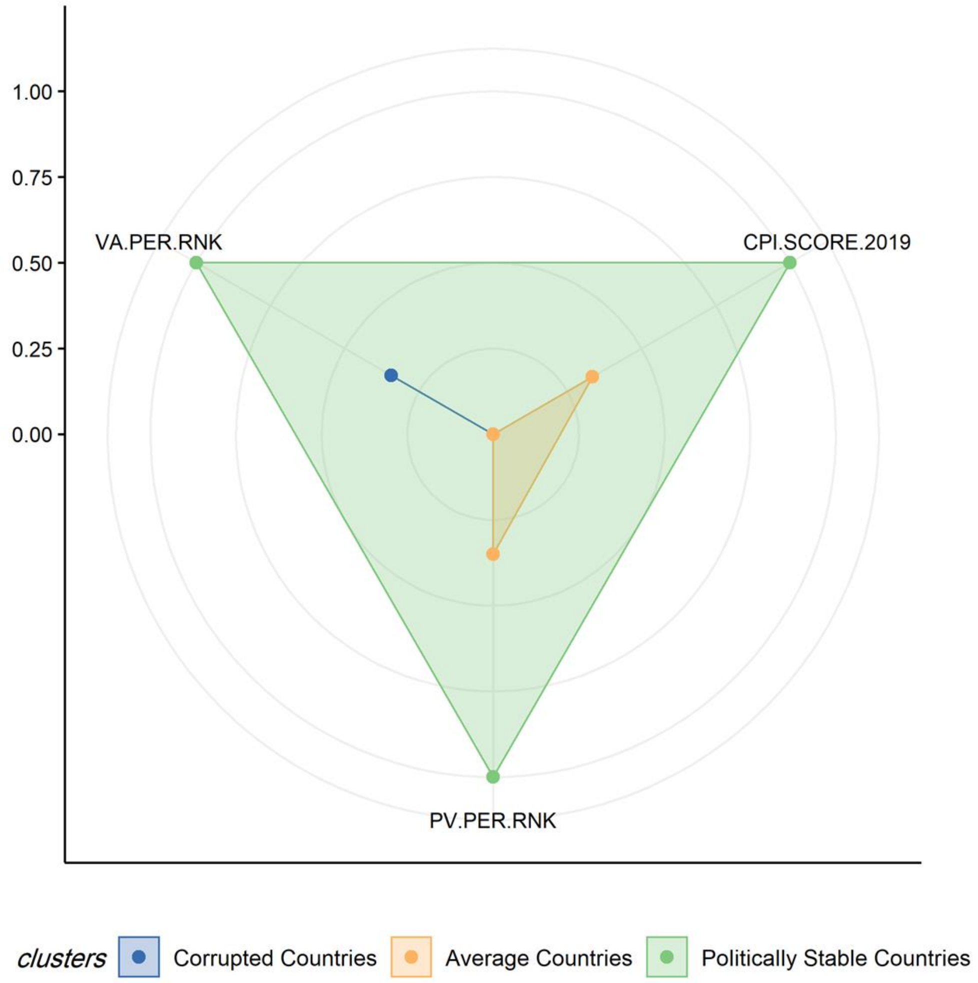

CPI.SCORE.2019: corruption perceptions index 2019
PV.PER.RNK: percentile rank of political stability and absence of violence/terrorism
VA.PER.RNK: percentile rank of voice and accountability

Figure S3: Radar Plot of the Clusters

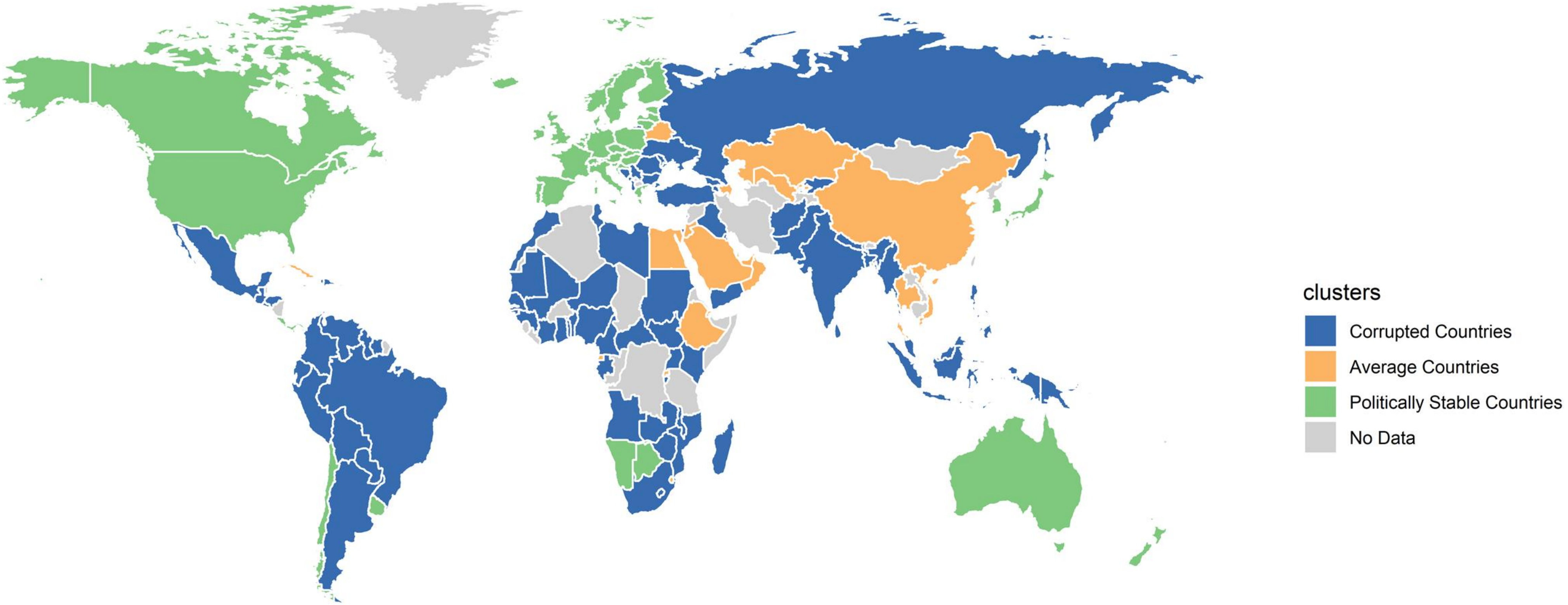

Figure S4: Distribution of Variables in Clusters (Model Based Clusters)
